# Anachronic Fruit Traits and Natural History Suggest Extinct Megafauna Herbivores as the Dispersers of an Endangered Tree

**DOI:** 10.3390/plants9111492

**Published:** 2020-11-05

**Authors:** Diego Muñoz-Concha, Karla Muñoz, Andrea P. Loayza

**Affiliations:** 1 Departamento de Ciencias Agrarias, Facultad de Ciencias Agrarias y Forestales, Universidad Católica del Maule, 3341695 Curicó, Chile; 2 Escuela de Agronomía, Facultad de Ciencias Agrarias y Forestales, Universidad Católica del Maule, 3341695 Curicó, Chile; k.munoz@hotmail.com; 3 Instituto de Investigación Multidisciplinario en Ciencia y Tecnología, Universidad de La Serena, 1720256 La Serena, Chile; aloayza@userena.cl; 4 Instituto de Ecología y Biodiversidad (IEB), 7800003 Santiago, Chile

**Keywords:** anachronism, megafaunal syndrome, seed dispersal, fruit ecology, *Gomortega keule*

## Abstract

Megafaunal seed dispersal syndrome refers to a group of traits attributed to the evolution of plants in the presence of large mammals. Present-day plants that bear these traits in areas where megafauna are absent are presumed to represent anachronic dispersal systems. *Gomortega keule* is an endangered tree species from a monotypic family (Gomortegaceae), endemic to Chile. Its fruit traits suggest adaptation to seed dispersal by large vertebrates; however, none are present today along its area of distribution. Here, we conducted a detailed revision on the fruit morphology of *G. keule* to examine whether its fruit traits fit a megafaunal dispersal syndrome. Additionally, we examined the fruit processing behavior of large domestic and captive wild animals fed with *G. keule* fruits, and its effect on germination. *G. keule* fruits had traits consistent with those of a Type 1 megafaunal fruit. Compared to intact, whole stones, seed germination probabilities decreased when fruits were handled by animals, suggesting that the seed was damaged during mastication and/or ingestion. Moreover, results from our feeding trials with elephants may also imply low efficiency of extinct gomphotheres as seed dispersers of this species. Our results also suggest that although domestic animals may disperse *G. keule*, it is unlikely that at present they can substitute the services of its original dispersers. Further investigation on seedling survival, local livestock management and forest management practices may help reinstate sexual regeneration in *G. keule*. Finally, integrating observations on fruit ecology and local people’s knowledge with experimental data enriches our species-centered approach and may help to address regeneration problems in other endangered plants.

## 1. Introduction

The idea of plant anachronisms refers to traits that appear to be the outcome of selective pressures imposed by past, rather than present-day ecological interactions [1]. The megafaunal seed dispersal syndrome is a compelling subset of these traits, represented by over-sized (>2 cm diameter) fleshy fruits encasing either a few large seeds (Type 1 fruit) or numerous small seeds (Type 2) [2,3]. In addition to their large size, other morphological characteristics of megafaunal fruits include indehiscence, seeds enclosed inside a strong protective casing (for Type 1 fruit), and fruits colored mostly in hues of brown, green or yellow [3].

Numerous plant species bearing megafaunal fruits occur in ecosystems that have long been deprived of large herbivores [4]; hence, they are presumed to be anachronic species presently dispersed by small vertebrates. Compared to their megafaunal counterparts, small animals disperse fewer seeds over shorter distances and do not ingest the seeds whole [5]. The loss of long-distance dispersal services provided by the megafauna has presumably lead to the restricted distributions of many megafaunal fruit plants, as well as reduced genetic flow among their populations (reviewed by Galetti et al. [4]). Additionally, if gut passage was necessary to stimulate seed germination, the loss of large herbivores may have also led to decreased germination and recruitment rates.

*Gomortega keule* is the only surviving species in an ancient lineage of the order Laurales in the Maulino and Valdivian forests of Chile, which bears fruits that appear to fit the megafaunal syndrome [6]; it is a large, yellow fruit with abundant edible flesh, firmly attached to a woody endocarp. There are no records of potential legitimate dispersers of this species, and little information regarding fruit consumption by extant vertebrates. *G. keule* has one of the largest fruits of Chile’s flora, yet despite the unique attributes of the species, a revision of the traits that support the megafaunal nature of its fruit is still lacking.

In this study, we provide detailed information on the fruit morphology of *G. keule*, comparing its fruit traits to those defined as megafaunal in the literature. Additionally, we report observations of handling and fruit processing behavior of large captive animals fed with *G. keule* fruits, as well as the results of a germination experiment using the stones processed by these animals. This experiment was conducted to test whether fruit handling treatment by present-day megafauna has an effect on *G. keule* germination. We use this information, coupled with natural history observations, to discuss whether *G. keule* constitutes a seed dispersal anachronism. We argue that a better understanding of the ecology of this species can help design more solid conservation plans, and that integrating observations on fruit morphology, ecology and local people’s knowledge with experimental data enriches our species-centered approach, and may also help to address regeneration problems in other endangered plants.

## 2. Results

### 2.1. Description of G. keule Fruits

**Figure 1:**
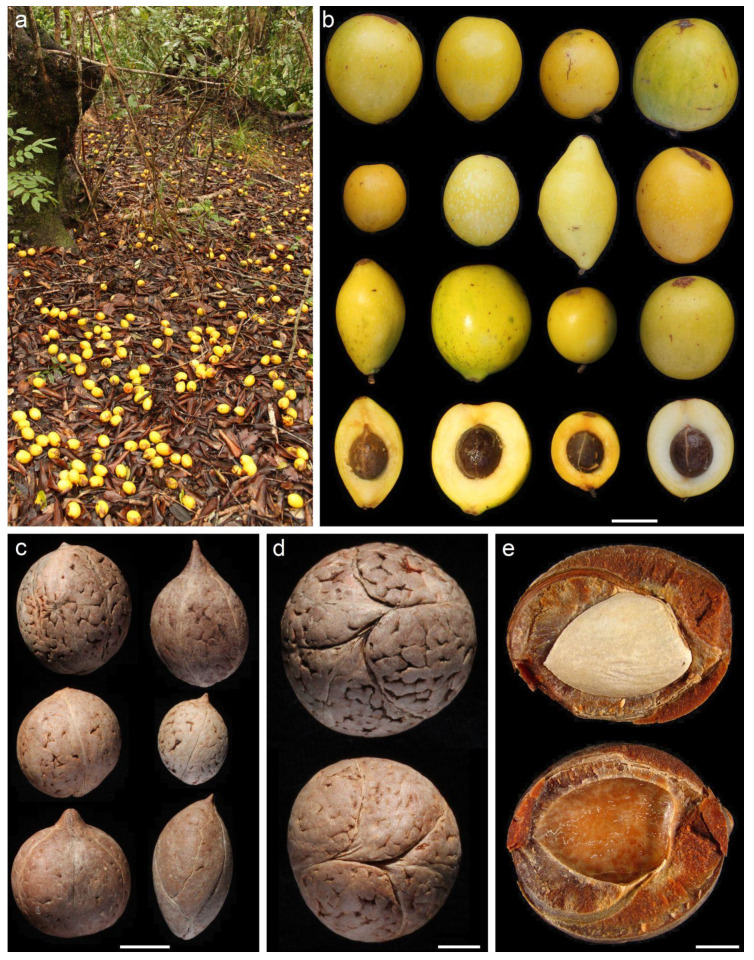
The fruit of *Gomortega keule*. (**a**) Fruits ripen and fall during the austral autumn and accumulated in large quantities on the ground due to the lack of consumption by extant native animals; (**b**) Morphological variation of *G. keule* fruits (bar scale = 3 cm); (**c**) Morphological variation of stones (bar scale = 1 cm); (**d**) Polar view of the stone showing the lines of carpel union (bar scale = 0.5 cm); (**e**) Open stone showing the hard thick lignified endocarp protecting the soft seed (bar scale = 0.5 cm).

The morphological attributes of *G. keule* fruits are shown in Table 1. The fruit corresponds to a rounded false drupe with a dense pulp or flesh. The surface of the fruit is glabrous and bright yellow (Figure 1a,b).

Stones are smooth, spherical to slightly elongated, and sometimes have a pointy end in the distal pole (Figure 1c). Normally, the fleshy pulp is strongly adhered to the endocarp. Stones typically have three lines from pole to pole corresponding to carpel union (Figure 1d); when a strong force is applied, the endocarp cracks and opens along these lines. Seeds are large, flat, soft, oily, and encased within the thick, woody endocarp (Figure 1e). In natural conditions, stones can remain many months, and possibly even years, on the ground and embedded in the forest litter, gradually losing hardness until they begin to open (D. Muñoz-Concha, Pers. Obs.).

Fruits are produced in sun-exposed branches, high in the canopy. They mature during the austral autumn and subsequently fall to the ground, where they accumulate in large quantities (Figure 1a). *G. keule* fruits have traits consistent with those of a Type 1 megafaunal fruit reported in the literature (Table 2); notably, oversized, yellow fruits with large seeds mechanically protected by a thick woody endocarp, and presented on the forest ground when mature.

### 2.2. In Situ Field Observations of Livestock Fruit Consumption

**Figure 2:**
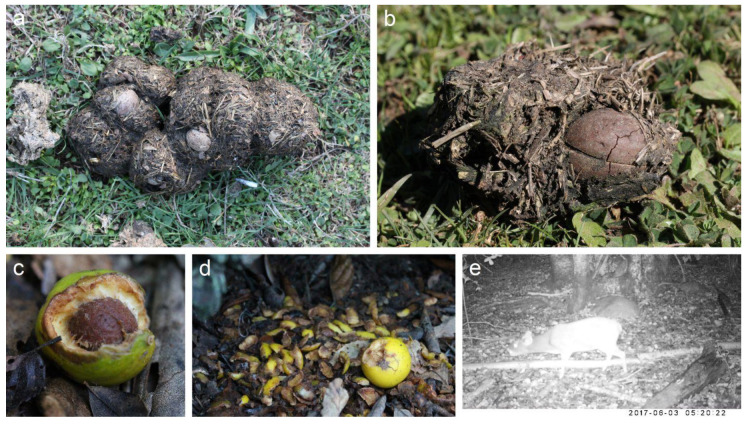
Evidence of consumption of *Gomortega keule* fruit by animals. (**a**) Two intact stones are visible in pig feces; (**b**) A stone showing an incipient crack in pig feces; (**c**) A fruit gnawed by rodents; (**d**) Gnawed fruit and flesh leftovers; (**e**) A cervid (*Pudu puda*) consuming the fruit flesh (image courtesy: Carlos Reyes and Alexis Villa—CONAF Maule).

At the Quile site, whole, intact stones were observed in cow and pig, but not horse feces, suggesting the former two are able to either swallow whole fruits or large chunks with the stone. A cracked stone was also observed in pig feces (Figure 2). During one year of monthly observations, no germination of *G. keule* seeds was observed at this location. However, in areas with native vegetation and less intensive cattle presence, we found several *G. keule* seedlings near conspecific trees.

### 2.3. Feeding Trials and Germination Experiments

Diverse behaviors were observed in zoo animals when presented with *G. keule* fruits. Red deer and alpacas did not approach fruits. The hippopotamus consumed fruits whole and their stones were retrieved two days later from the water pool where the animal defecated. Elephants exhibited two distinct behaviors with *G. keule* fruits: (1) they could munch the fruits and discard the stones, or (2) they could ingest the whole fruit (observed only during the second year of feeding fruits to elephants). In captivity, pigs consumed the fruit flesh, but did not ingest the stone. Cows and horses ate the fruit flesh, discarding the stone.

**Figure 3:**
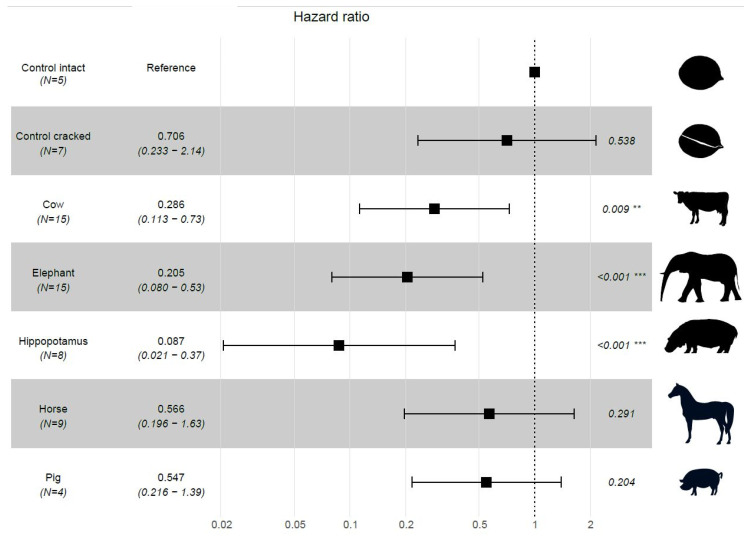
Forest plot of the Cox proportional hazards regressions of germination clustered by pot replicate (*N*). The plot shows how the seed germination hazard ratio (HR) and 95% CI from stones processed by each of the animal species studied and from cracked stones compares to the HR of whole, intact stones. The germination HR of a seed from an intact stone is standardized to 1 and denoted by the dashed vertical line. An HR > 1 indicates an increased germination probability, whereas an HR < 1 indicates a decreased probability.

The percentage of seed germination from intact stones was 72.0 ± 30.3 (mean % ± SD), 60.0 ± 25.8 from cracked stones, 45.3 ± 22.0 from stones discarded by cattle, 37.3 ± 22.5 for those discarded by elephants, 15.0 ± 23.3 for stones in hippopotamus feces, 64.4 ± 37.1 for stones discarded by horses and 70.0 ± 20.0 for those discarded by pigs. These differences translated into different germination hazard ratios (Waldχ^2^ = 40.39, df = 6, *p* < 0.001); specifically, the germination hazard ratio (i.e., the ratio of germination between each treatment and the control group) of seeds in stones processed by elephants, cows and the hippopotamus was significantly lower than that of seeds within intact whole stones (Figure 3), suggesting that the seed is damaged when these animals process the fruit.

## 3. Discussion

Here we show that the morphological attributes of *G. keule*’s fruits correspond to those defined as megafaunal in the literature. Since there are no contemporary large (>50 kg) mammals co-occurring with this species, its fruit traits may have evolved for seed dispersal by the extinct megafauna of the Pleistocene. Field observations and accounts from local people, reveal that in the present-day livestock consume *G. keule*’s fruits; however, results from the feeding trials suggest that the seeds of some of the stones discarded by these animals are damaged during mastication, resulting ultimately in decreased germination. Overall, our results suggest that it is unlikely that livestock is performing the seed dispersal services formerly provided by the megafauna.

### 3.1. Do G. keule Fruits Bear the Traits of a Megafaunal Dispersal Syndrome?

The megafaunal seed dispersal syndrome refers to extant plants that bear ‘overbuilt’ fruits, too large to be dispersed by present-day herbivores and are thus presumably adapted for seed dispersal by the megafauna that became extinct at the end of the Pleistocene [1]. In addition to fruit size, the definition of what constituted megafaunal fruit traits remained vague and controversial until Guimarães et al. [3] introduced an operational definition and a set of criteria that allowed a classification of megafaunal fruits based on their traits. Using these criteria, the fruit of *G. keule* is a Type 1 megafaunal fruit based on size, indehiscence, presence of large seeds, and fruit color.

Type 1 megafaunal fruits are distinguished by having one to five large seeds [2]; *G. keule* generally has one seed per fruit. Seed hardness is an additional characteristic of megafaunal fruits identified by Feer [2]. The mechanical protection of the plant embryo is a requisite to successfully travel through the digestive system of an animal without being damaged. *G. keule* seeds are protected by a thick and tough endocarp that can reduce seed damage during chewing and during passage through the digestive tract.

Finally, green, brown and yellow fruit colors were identified by Guimarães et al. [3] as the most prevalent colors in megafaunal fruits. *G. keule* fruits are bright yellow when ripe, but can also occasionally be greenish (Figure 1b).

Overall, fruit traits of *G. keule* strongly suggest a megafaunal seed dispersal syndrome, and since there are no extant megafaunal mammals (>50 kg) in its area of distribution, this species may represent a seed dispersal anachronism. Nonetheless, it is possible that these traits may have also partly resulted from phylogenetic inertia [9], as the vast majority of species within the Laurales have large fruits that are animal dispersed [10], and all members of the sister clade of Gomortegaceae (i.e., Siparunaceae) possess drupaceous fruits [11].

### 3.2. Do Extant Co-Occurring Native and Domestic Mammals Act as Dispersers of G. keule?

Due to their large size relative to extant native species, it has been suggested that livestock may act as contemporary seed dispersers of megafaunal fruit plants [1,3]. In our study area, local people report that livestock consume *G. keule* fruits. Pigs and sheep typically discard the stones while chewing, which is consistent with the high densities of defleshed stones observed under fruiting trees. However, presence of stones in pig feces also revealed that these animals can ingest whole fruits or big fruit chunks with the stone attached (Figure 2). With respect to cows, people recount that they spit stones when they lay ruminating, leaving stone piles at their resting places. We could not confirm this observation with empirical data; nonetheless, in central Chile, cows exhibit the same behavior with *Jubaea chilensis*, another megafaunal fruit plant (A. Loayza, Pers. Obs.). Moreover, a recent metanalysis [12] revealed that ruminant animals frequently regurgitate large hard seeds during rumination, which reinforces the potential role of members of this group as dispersers of megafaunal plants. Cows can also swallow whole fruits, as revealed by the presence of stones in their feces, but it is unknown which of the two fruit processing behaviors is more common.

Although livestock consume *G. keule* fruits, it is uncertain whether they provide effective dispersal services. Several factors need to be considered to ascertain their role as potential substitute dispersers of this species. Among these are the distance to which they can disperse the stone, the suitability of the deposition sites for seedling establishment and, whether they damage the seed during fruit processing. It is unlikely that at present livestock provide long-distance seed dispersal services, as livestock typically feed from the large amounts of fruits that have fallen on the ground [1], discarding the stones at the site of encounter. This behavior, coupled with the lack of other dispersers, results in seedling recruitment close to parent plants, deriving into spatially aggregated genetic neighborhoods [13]. There is no information regarding other habitats where livestock deposit the stones, nor of their suitability for seedling establishment; thus, future studies should address these issues. Finally, not all livestock have equal roles as dispersers with respect to their effect on germination [14]. Here, we show that cows likely damage the seed during mastication, which results in lower *G. keule* germination; in contrast, fruit processing by pigs and horses does not appear to damage the seed, making them potentially better dispersers than cattle. Currently, there is a large knowledge gap regarding which livestock species can act as surrogate dispersers of megafaunal fruit plants, and how they may differ regarding their dispersal effectiveness. In Chile, there are several relict plant species with megafaunal fruits, but no large or native animals that could provide long distance-dispersal services; therefore, it is of key interest to understand the potential role that livestock play in their dispersal.

There is very little information regarding fruit consumption of *G. keule* by native animals. There is some evidence that a small native deer (*Pudu puda*) consume the pulp of the fruit (Figure 2e), but given its small size (8–10 kg), it is unlikely that it can swallow the stone. Rodents are also known to consume *G. keule* fruits; they appear to consume mainly the pulp, leaving the stone intact (Figure 2c,d). A recent study revealed that *Rattus rattus* remove *G. keule* fruits, but the fate of the seeds in this case is unknown [15]. In this study, the authors reported several native rodents in their study site, but none interacted with the fruit of *G. keule*. The role of rodents as dispersers for megafaunal species in the Neotropics has been demonstrated by several authors [1,16,17,18]; thus, further research is needed to understand their role as potential dispersers of *G. keule*.

### 3.3. Can the Seed of G. keule Be Dispersed by Present-Day Large Animals?

Our results from the feeding trials revealed that not all animals consume *G. keule* fruits and furthermore, that fruit processing behaviors of ex situ domestic animals are different from those living in natural areas with *G. keule* trees. The manner by which animals processed fruits while feeding had an effect on seed germination. Overall, mastication negatively affected germination, since animals that exhibited this behavior (i.e., cattle, elephants and the hippopotamus) led to reduced germination rates. This suggests that the bite force of these animals can alter the protective woody shell of *G. keule* and damage the seed. Therefore, although cattle can disperse *G. keule*, this interaction comes with a cost represented by a lower seed germination. Our results regarding the effect of stone mastication by elephants may shed some light as to the possible effects of gomphothere (*Stegomastodon platensis*; [19]) dispersal for *G. keule* germination. The dentition of gomphotheres suggests they were forest browsers, which broke down food mechanically by a combination of grinding and shearing [20]. Consequently, it is likely that they also damaged the seed during mastication, resulting in a relatively low dispersal effectiveness of *G. keule*. This, however, could have been partly compensated by the large quantities of fruits they presumably consumed.

In this study, we found that seed germination from stones spat out by horses and pigs is comparable to that from intact *G. keule* stones, revealing that these animals do not damage the seed if they do not swallow the stone. This suggests that horses and pigs could serve as potential present-day dispersers of *G. keule*, as they do for other plant species with a megafaunal syndrome [21,22]. These results also give insight into the potential role of Pleistocene equids as dispersers of this species. Overall, our results may suggest that although a portion of the seeds were damaged during feeding, livestock can disperse *G. keule* seeds.

## 4. Materials and Methods

### 4.1. Study Species

*G. keule* is an evergreen tree belonging to the monotypic family Gomortegaceae. It is endemic to a narrow area of the coastal mountain range in south-central Chile, originally associated with deciduous forests dominated by *Nothofagus* species. Today, large portions of these forests have been replaced by plantations of exotic tree species, resulting in fragmented *G. keule* populations [23]. Currently, this species is restricted to 25 populations [24] and listed as endangered by the IUCN [25].

*G. keule*’s fruit is a fleshy, yellow false drupe, encasing typically one, and occasionally two or three soft and oily seeds, within a hard, woody endocarp [6], which is difficult to open; moreover, this action often damages the seed (D. Muñoz-Concha, Pers. Obs.). The stone, comprised by the seed and the endocarp, constitutes the unit of dispersal.

Seed germination, which is recognized as an important factor influencing the conservation status of the species, is highly variable (i.e., 4–42%) (reviewed by Muñoz-Concha and Davey [6]). Germination times are long (up to 18 months), and the hard endocarp appears to act as a mechanical barrier [6].

### 4.2. Traits of Gomortega keule and In Situ Field Observations

We measured morphological attributes from 2538 fruits collected from 2012 to 2019, from 55 trees distributed in three localities: Ralbún (36°03′51” S; 72°38′29” W), Quile (36°02′33” S; 72°42′25” W), Copiulemu (35°59′56” S; 72°40′40” W) and Los Queules National Reserve (35°59′15” S; 72°41′43” W). Measurements included equatorial diameter, polar diameter, whole fruit mass, and stone mass. To examine whether *G. keule* fruits fit the megafaunal dispersal syndrome, we contrasted the trait attributes obtained to those defined as megafaunal trait attributes in the literature.

To gather information on how domestic animals interact with *G. keule* fruits, we conducted in situ field observations in natural stands of *G. keule* in Quile, a private forestry farm with minor agriculture and livestock production. Of special interest were the visits in 2020 to an enclosure (ca. 0.5 ha), which included three *G. keule* trees that fruit each year. During the visits, we inspected livestock feces for the presence of stones.

### 4.3. Feeding Trials and Germination Experiments

In June and July 2012, we collected fruits from Ralbún and transported them to Santiago, where they were presented as the first meal of the day to four large (>45 kg) vertebrate species at the Zoológico Nacional (Chile), the only zoo in the country with elephants. Fruits were also presented to four livestock species in three farms near Curicó, Chile (Table 3). Each animal was offered 100 fruits, and we collected stones from all fruits that were handled by each of the species; that is, gut-passed stones from feces, as well as stones that were spat out during feeding.

To determine if fruit processing by the different animal species had an effect on germination, all stones retrieved per species were pooled and then placed in groups of five in pots filled with a compost substrate. Please note that with the exception of stones processed by the hippopotamus, which passed through its digestive tract, all stones used for the experiment were spat out after pulp consumption by each of the animal species examined. We established 4–15 replicates (i.e., pots) per species. We followed the same procedure to set up two control treatments: intact stones (five pots) and cracked stones (seven pots). The latter represents either the possible chewing effect of an animal, or the expected weakening of the mechanical barrier. Pots were seeded in June 2012 and seed germination was monitored weekly during two years by digging out the stones. We assessed whether the temporal patterns of germination differed among groups using Cox Proportional Hazards models for censored data clustered by pot to account for non-independence. In these models, we used seed germination of intact seeds as the standard for comparison, since it represents the most common present-day state of *G. keule* seeds in natural conditions. Statistical analyses were performed with the R statistical environment using the *survival*, *ggfortify* and *survminer* packages [26,27,28,29].

## 5. Conclusions

The most likely original seed dispersers of *G. keule* were large animals, such as equids and ground sloths, which inhabited South America until the end of the Pleistocene. Gomphotheres may have also dispersed its stones; however, it is likely that many of the seeds were damaged during mastication. The extinction of megafauna had consequences for the plant species they dispersed, disrupting long-distance seed dispersal, decreasing establishment, reducing gene flow among populations and its geographic range [4]. In this sense, the loss or strong reduction of long distance dispersal may explain the high level of genetic differentiation among *G. keule* populations [30,31], even those separated by only a few kilometers [32]. The survival of *G. keule* from the end of the Pleistocene to present day may be explained by its high ability to propagate vegetatively [32], the germination of seeds near parent trees [13], and occasional dispersal by other species. However, increasing fragmentation of its habitat and anthropogenic disturbances are imposing further challenges to the recruitment of this endangered species.

The set of traits observed in the fruit of *G. keule* fits well within the megafaunal syndrome, suggesting that this tree evolved in the presence of large animals acting as the main seed dispersal agents. Although some domestic animals today ingest the fruit, it is unlikely that they can substitute the services of the original disperser, because they damage the seed during fruit processing, or because the seeds are not being deposited in suitable areas for their recruitment. Moreover, the high germination rates contrast with the scarcity of in situ seedlings, suggesting that other factors are limiting seedling establishment. However, it is possible that further knowledge of seedling survival, local livestock management and forest management practices, may achieve sexual regeneration reinstatement and thus reactivate the genetic flow and adaptation dynamics of the species, with important consequences for its conservation.

## Figures and Tables

**Table 1:** Measurements of *Gomortega keule* fruits.

Trait	Mean	Standard Deviation	n	Maximum	Minimum
Fruit equatorial diameter (mm)	33.11	4.82	2538	53.2	21.0
Fruit polar diameter (mm)	42.55	7.53	1977	71.6	20.9
Fruit weight (g)	30.23	10.33	2344	78.4	6.2
Stone weight (g)	6.42	1.63	1903	12.4	1.6
Stone proportion (% of fruit weight)	21.42	5.54	1902	48.0	7.7

**Table 2:** Type 1 fruit traits associated with seed dispersal by megafauna exhibited by *Gomortega keule*.

Propagule Trait	Presence in *G. keule*	Species Reported
Fruit generally > 2 cm diameter	Yes	49 species in 8 families [3]
Big seeds	Yes	49 species in 8 families [3], *Klainedoxa gaboensis*, *Panda oleosa*, *Gambeya lacourtiana* [2]
Strong seed coat	Yes	*Balanites* spp. [7], *Sclerocarya birrea* [8]
Dull colour: green, brown, yellow	Yes	*Balanites maughamii*, *Sclerocarya birrea* [8]
Scented fruit	No	*Balanites maughamii* [8]
Mature fruit presented on ground	Yes	*Balanites maughamii*, *Sclerocarya birrea* [8]

**Table 3:** Animal species presented with fruits of *Gomortega keule*. All species were presented 100 fruits. The table shows whether and how animals handled the fruits.

Species		N° of Animals	Observations
Pig	*Sus scrofa*	20	Consumed flesh partly, did not ingest the stone.
Cow	*Bos taurus*	3	Consumed flesh, did not ingest the stone.
Horse	*Equus caballus*	3	Consumed flesh, did not ingest the stone.
Sheep	*Ovis aries*	3	Consumed flesh, did not ingest the stone.
Elephant	*Loxodonta africana*	2	Consumed flesh discarding the stone (sometimes cracked). Second year ingested.
Red deer	*Cervus elaphus*	1	Did not approach the fruits.
Alpaca	*Vicugna pacos*	1	Did not approach the fruits.
Hippopotamus	*Hippopotamus amphibius*	1	Consumed the whole fruit.
